# Chronic Auditory-Nerve Implant Enhances Brainstem Phase Locking to Electric Pulse Trains

**DOI:** 10.1007/s10162-025-01003-1

**Published:** 2025-08-14

**Authors:** John C. Middlebrooks, Matthew L. Richardson, Robert P. Carlyon, Harrison W. Lin

**Affiliations:** 1 Department of Otolaryngology - Head and Neck Surgery, University of California, Irvine, CA USA; 2 Cambridge Hearing Group, MRC Cognition & Brain Sciences Unit, University of Cambridge, Cambridge, England

**Keywords:** Cochlear implant, Temporal fine structure, Pitch, Cat, Frequency following Response, Intraneural stimulation, Auditory nerve electrode

## Abstract

**Purpose:**

Present-day cochlear implants (CIs) can deliver usable speech reception in quiet surroundings. Most CI users, however, show impaired sensitivity to temporal fine structure, which hampers their use of pitch contours and spatial cues to segregate competing talkers. In previous short-term animal studies, we used intraneural (IN) electrodes to stimulate pathways originating from various cochlear turns. Neurons in the inferior colliculus synchronized to apical stimulation at higher rates than to stimulation of the middle-to-basal pathways that are stimulated primarily by today’s CIs. Here, we use non-invasive recordings to test the safety and efficacy of up to 6 months of IN implantation and stimulation in cats.

**Methods:**

Deafened cats (ten female, two male) were implanted with IN and/or conventional CI electrodes. The IN electrodes were single activated-iridium shanks that targeted apical-turn fibers. Scalp recordings were made from sedated animals at 2–3-week intervals. Auditory brainstem responses to single electrical pulses (eABR) tracked sensitivity and growth of responses. Frequency following responses to electrical pulse trains (eFFR) assessed brainstem temporal transmission at varying pulse rates.

**Results:**

Thresholds for eABR were lower for IN than for CI stimulation, dynamic ranges were wider, and (by inference) spread of activation was more restricted. The eFFR evaluated at latencies comparable to those of inferior-colliculus spikes synchronized at maximum pulse rates averaging > 360 pulses/s for IN compared to ~ 240 pulses/s for CI stimulation. The eABR thresholds and eFFR cutoff rates were stable out to 6 months after implantation.

**Conclusions:**

The results demonstrate the safety and efficacy of chronic IN stimulation in an animal model. In a future clinical device, an IN electrode could augment cochlear-implant performance by enhancing temporal acuity, thereby improving speech reception amid competing sounds.

## Introduction

Most present-day cochlear-implant (CI) users can expect to achieve usable speech reception in quiet surroundings. Nevertheless, nearly all CI users struggle to understand speech in everyday noisy conditions, such as in restaurants, busy offices, or classrooms [1, 2]. Normal-hearing listeners can use pitch contours and spatial source separation to segregate a stream of syllables from one talker from those of other talkers [3]. Both pitch perception and spatial hearing, however, rely on sensitivity to temporal features of sound, and this sensitivity is markedly impaired in CI users, particularly at high electrical pulse rates. Most CI users, for instance, are unable to rank the pitches of electrical pulse rates higher than ~ 300 pulses per second (pps) [4, 5] and bilaterally implanted listeners show essentially no useful sensitivity to interaural time differences at these higher rates [6]. Arguably, the poor speech reception of CI users in everyday complex auditory scenes can be traced to the limited sensitivity of users to temporal fine structure (TFS) [7].

A possible explanation for the limited TFS sensitivity of CI users has been demonstrated in an animal model [8–10]. In those non-survival experiments in anesthetized cats, the auditory nerve was stimulated with electrical pulse trains, either with an array of electrodes in the cochlear scala tympani, like a clinical CI, or with a novel intraneural (IN) electrode that penetrated the trunk of the auditory nerve as it exited the basal cochlear turn; the IN device used in those studies had 16 sites that could excite restricted fiber populations from throughout the cochlear spiral. Electrically evoked single- and multi-unit activity was recorded with microelectrodes from the central nucleus of the inferior colliculus (ICC). Results showed that, compared to CI stimulation, IN stimulation produced thresholds lower by about 25 dB, narrower tonotopic spread of excitation, and broader dynamic ranges [8, 9]. Notably, temporal acuity was enhanced such that more than twice as many ICC neurons synchronized to electrical pulse rates > 300 pps delivered from IN stimulation compared to that from CI stimulation [10]. The enhanced temporal acuity turned out not to be a special property of IN stimulation per se. Rather, enhanced temporal acuity was limited to stimulation of apical fibers that projected to ICC neurons having characteristic frequencies (CFs) lower than ~ 1.5 kHz. Middle-to-basal-turn stimulation with either IN or CI electrodes, activating higher-CF pathways, showed temporal transmission limited to lower rates, with no clear systematic variation of temporal acuity across CFs higher than ~ 2 kHz (at least in cats; shown in Figs. 6 and 7 of reference [10]). Presumably, selective stimulation of apical fibers activated low-CF brainstem pathways that are adapted for high temporal acuity. Those high-acuity pathways are not stimulated selectively by today’s CIs, which lie mostly in the basal and middle cochlear turns and which stimulate apical cochlear neurons only broadly, if at all.

The short-term experiments in animals raised the possibility that IN stimulation could enhance the sensitivity of human CI users to TFS. Pursuant to that goal, we now have tested the safety and efficacy of up to 6 months of chronic IN implantation in an animal study. We specifically tested the hypotheses that, compared to CI stimulation, IN stimulation of apical fibers would activate the auditory pathway at lower thresholds and would elicit stimulus-synchronized activity in the auditory midbrain at higher rates. The IN device used in the previous short-term experiments was a thin-film silicon substrate supporting 16 electrode sites. It was uncertain whether such a device would be stable over the decades needed for a clinical prosthesis. For that reason, we employed an IN device consisting of a single iridium shank, sharpened, insulated except at the tip, and activated by deposition of an oxide layer. We implanted cats chronically with the IN electrodes and/or conventional CI electrode arrays. Scalp recordings of the electrically evoked auditory brainstem response (eABR) yielded the sensitivity and growth of response magnitude of the auditory pathway. The frequency-following response (eFFR) provided a measure of synchronized activity at various levels of the pathway. Recordings from sedated cats were repeated at ~ 2–3-week intervals for as long as 6 months.

We envision future compound clinical devices that would offer the good-to-excellent speech-recognition performance of today’s CIs augmented by an IN electrode providing high-rate transmission of TFS. Here, we tested a cohort of animals with a “CI + 1” device consisting of a 7-channel animal version of a clinical CI plus a single IN electrode implanted in the same ear. The results from CI and IN components of the CI + 1 devices are included here along with the results from CI or IN devices alone.

## Methods

### Overview

We tested electrophysiological responses to IN electrodes that were implanted in the auditory nerves of cats for as long as 6 months. Activation of the auditory pathway and transmission of TFS was monitored by scalp recording of eABR and eFFR at ~ 2–3-week intervals. For comparison with the IN stimulation, data also are presented here from a cohort of cats that had CIs chronically implanted in their scalae tympani [11]. A third cohort of cats was implanted in the present study with “CI + 1” devices consisting of both IN and CI electrodes in the same ear. Throughout, the principal analyses were comparisons of IN versus CI stimulation, irrespective of the specific cohorts. The animal work is reported following ARRIVE guidelines.

### Animals

All procedures were conducted in accordance with the National Institute of Health guidelines and with the approval of the Institutional Animal Care and Use Committee for the University of California at Irvine. Domestic short-hair cats (*Felis catus*) were obtained from a breeding colony at the University of California at Davis. Results are presented from ten females and two males that ranged in age from 0.9 to 6.6 years (median 1.8 year) and in weight from 2.5 to 4.7 kg (median 3.4 kg) on the day of implantation. NIH-approved group housing was employed. The two male cats (Cats NU and FO) and two of the females (SC and BR) were neutered to facilitate group housing. Chronic implantations of IN electrodes alone were tested in five cats (BO, NO, BU, AP, SC). An additional four cats were tested with CI + 1 devices that comprised both IN and CI devices in the same ear; those animals are denoted in the figures by filled symbols. Two of those CI + 1 cats (BR, FO) yielded both CI and IN data. In the others (AB, ED), the IN electrode failed due to technical problems, but usable data were obtained from the surviving CI arrays. An additional three cats were tested with chronic CIs alone in another study from our lab [11] (Cats MO, NU, XE). Altogether, the 12 cats yielded results from chronic study of seven IN electrodes (denoted in the figures by blue color and solid lines) and from the most apical electrodes of seven CI arrays (denoted by red color and dotted lines).

### Implanted Electrodes

**Fig. 1:**
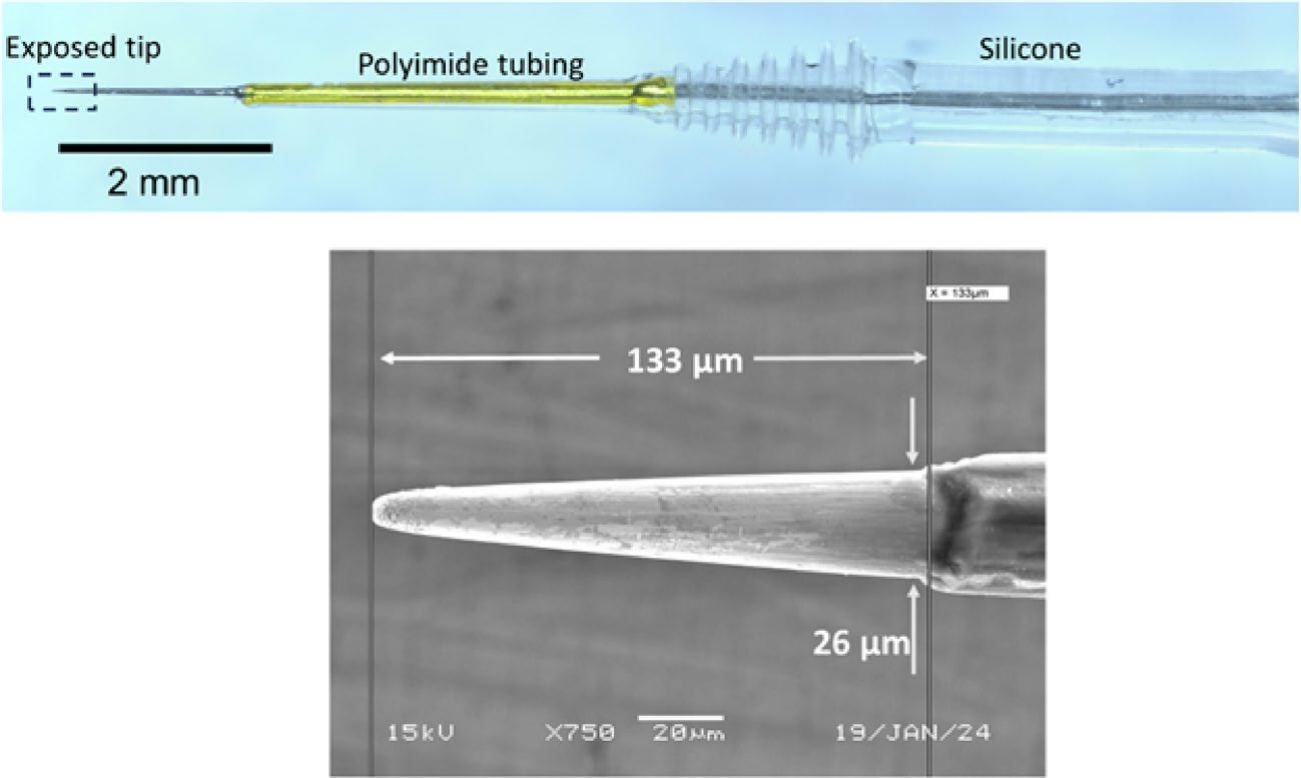
The intraneural electrode. The optical image on the top shows the tapered iridium shank, stiffened near the tip with polyimide tubing, and bolstered by a silicone gripping surface. The scanning electron micrograph on the bottom shows the exposed iridium oxide tip. The Parylene-C insulation is visible near the right edge of the image

The intraneural electrodes were iridium shanks, 100 µm in diameter, tapered to a ~ 6–8-µm-diameter tip and insulated with Parylene-C (Microprobes for Life Science, Gaithersburg, MD) (Fig. 1). The exposed tips were ~ 5000 µm^2^ in geometric area. The tips were activated by Advanced Bionics, LLC (Valencia, CA) by depositing an oxide layer with use of cyclic voltammetry. The activation increased the charge injection capacity of the electrode surface, thereby decreasing the active impedance and reducing the danger of electrotoxicity to neural tissue. Advanced Bionics also added a molded silicone gripping surface to the IN devices.

The intrascalar CIs implanted chronically in this and the previous study [11] were 8-channel lateral-wall research electrode arrays produced by Advanced Bionics. The electrodes were spaced at 0.75-mm intervals. The electrode material was platinum, and the surface area of each half-cylinder electrode was ~ 110,000 µm^2^. Only the most apical electrode in each array was used in the results reported here because that electrode had the tightest fit in the scala tympani, it generally had the lowest threshold, and it was the most comparable to the IN electrode in regard to the CFs of stimulated fibers. The return electrode for both IN and CI devices was a platinum cylinder, 1.5 mm in diameter and 1.6 mm long. The IN or CI electrodes and return electrodes each were attached to flexible coiled cables leading to a 9-contact percutaneous connector.

The CI + 1 devices combined one CI array and one IN electrode. They differed from individual CI and IN electrodes only in that the cables of both devices connected to a single 9-pin percutaneous connector, which served the most apical seven of the CI electrodes, a single IN electrode, and a single return electrode. The CI and IN components of the CI + 1 devices were tested in the same sessions but in separate series of trials; they never were stimulated simultaneously. In the CI + 1 cohort of four cats, there were technical problems with the IN electrodes in two of the animals. For that reason, we present results from only the two successful IN electrodes from that cohort (in Cats BR and FO). Notably, there was no indication of impairment of responses to the CI electrodes associated with failure of the IN electrodes. For that reason, we present results from the most apical CI electrodes in each of the 4 CI + 1 animals; that includes the apical CI electrode in Cat ED even though it was lost after test day 72 due to fouling of the connector.

### Deafening and Chronic Implantation

With two exceptions, cats were deafened bilaterally 2 weeks prior to implantation using systemic administration of a single dose of an ototoxin (kanamycin, subcutaneous, 300 mg/kg) and a diuretic (ethacrynic acid, intravenous) [12, 13]. The ethacrynic acid was given in successive doses at 10-min intervals until no ABR to sound could be observed at equipment maximum, ≥ 65 dB above each cat’s normal-hearing threshold; final doses of ethacrynic acid ranged from 10 to 23.4 mg/kg, median 13.2. Of the two exceptions, one (Cat ED) showed signs of acoustic hearing on the day of implantation prompting the deafening procedure to be repeated, and for the other (Cat FO), deafening was postponed until 60 days after implantation to permit some psychophysical tests with acoustic hearing. We observed no obvious difference among animals in responses to electrical stimulation before and after deafening or re-deafening.

Each cat was implanted with an IN electrode and/or a CI array using sterile procedures with isoflurane gas anesthesia. Implantation of the device(s) began with exposure of the tympanic bulla. The bulla was opened with a carbide burr, and the round window was visualized. For implantation of the IN and/or the CI + 1 devices, the round window margin was enlarged with a diamond burr. The CI was inserted through the round window, until the most apical electrode lodged in the scala tympani, about 8–9 mm deep to the round window margin. For the IN implantation, the osseous spiral lamina of the basal cochlear turn was visualized through the round window and a ~ 50 µm-diameter hole was made using the beveled tip of a 30-ga needle. As in our previous studies [8, 10], that approach targeted the trunk of the auditory nerve as it exited the basal turn. The IN electrode was held with a custom forceps positioned by a micropositioner. The forceps had a 3-cm offset near the tip that permitted visualization of the electrode unimpeded by the micropositioner. The IN electrode was advanced through the osseous spiral lamina toward the trunk of the auditory nerve, basal to the basal cochlear turn. The electrode was advanced until it penetrated the epineurium, entered the nerve, and elicited eABR waveforms in response to single electrical pulses. In the anatomical study by Arnesen and Osen (1978) and in our previous study of IN stimulation [8], those superficial layers of the nerve consisted of fibers from the cochlear base that activate high-frequency brainstem pathways. The electrode then was advanced another 500 or 750 µm to the center of the nerve, which is the typical location of fibers from the cochlear apex [8, 14]. In a few instances, advances of the electrode led to an increase in the eABR threshold, whereupon the electrode was retracted slightly.

The CI and/or IN devices were secured by filling the bulla with acrylic cement. The return electrode was inserted in deep neck musculature caudal to the bulla. Cables for the stimulating and return electrodes were led under the temporal muscle to the dorsal midline of the skull, where the percutaneous connector was fixed in place with skull screws and acrylic cement. The connector was protected by a stainless-steel cylinder that was fixed to the skull. The cylinder was covered with a screw-on cap, and the surgical incision was closed around the cylinder.

### Electrical Stimulation and Scalp Recording

Stimulus generation and data acquisition used hardware from Tucker-Davis Technologies (TDT; Alachua, FL) controlled by custom MATLAB scripts (The Mathworks; Natick, MA) on a Windows-based personal computer. Electrical stimuli were generated by a TDT IZ2H optically isolated current-controlled stimulator. The output sample rate was 24,414 samples/s. Scalp recordings were made with a TDT Medusa4Z amplifier, also at 24,414 samples/s. Signals were bandpass filtered on-line between 1 and 4000 Hz and saved to computer disk for off-line analysis.

All stimuli consisted of electrical pulses, either single pulses or as trains of pulses. Each pulse was symmetrical, biphasic, 82 µs per phase, with no inter-phase gap. Cathodic- and anodic-leading pulse polarity alternated between trials for the purpose of reducing electrical artifact in the online averaged display.

Impedances of IN and CI electrodes were measured in situ during each test session using a 6-kHz sinusoidal probe signal. The period of that frequency (166.7 µs) was close to the sum of the phase durations of the biphasic pulses (2 × 82 µs). At ~ 10 weeks post-implantation, impedances of the IN electrodes measured in the auditory nerve ranged from 3.4 to 8.0 kOhm (mean = 6.3 kOhm). Impedances of the most apical CI electrode in each array ranged from 5.1 to 12.4 kOhm (mean = 8.2 kOhm). On average, the IN and CI electrodes showed no significant difference in impedance (*t* = 1.5,* p* = 0.17, *df* = 12). Issues of IN and CI electrode surface area, material charge-injection capacity, and impedance are considered in the “Discussion” section; briefly, differences in the charge-injection capacity of the electrode materials (iridium oxide for IN versus platinum for CI) compensated for the differences in electrode surface area.

Scalp recordings from the implanted cats began ~ 2 weeks after implantation and were repeated at ~ 2–3-week intervals. Cats were sedated for scalp recording with ketamine (25 mg/kg, i.m.) and acepromazine (1 mg/kg, i.m.). The sedation was intended to minimize spontaneous movements and to block any discomfort from the transdermal recording electrodes. Cats typically were areflexic shortly after the onset of anesthesia but sometimes showed weak corneal-blink and/or limb-withdrawal reflexes 1/2 to 1 h after the initial injection. Supplementary injections of ketamine alone were administered if the cats showed spontaneous movements.

The eABR and eFFR were recorded from the scalp with transdermal electrodes. The electrodes were hypodermic needles, polished with abrasive to remove any silicone coating. The needles were inserted through the skin. Ipsilateral and contralateral recordings were made simultaneously with working leads placed over each mastoid, respectively ipsilateral and contralateral to the stimulated cochlea. The common reference electrode was on the midline of the scalp, rostral to the percutaneous connector. Waveforms were inverted offline to that they are illustrated with vertex positivity (i.e., positivity at the midline reference electrode) shown as upward. Waveforms recorded with a particular electrode laterality varied among animals, probably due to variation in the distortion of current paths by the skull cylinder and its acrylic cement. In some cases, the position of the contralateral working lead was adjusted to strengthen recording of the waveform displacement having a latency of ~ 5 ms, which we attribute to an auditory-midbrain generator. Generally, eABR thresholds were lower ipsilaterally because low-threshold responses by lower brainstem structures were more prominent in those recordings. Ipsilateral recordings were used for quantitative measures of eABR thresholds. Activity at latencies that corresponded to midbrain activity was stronger contralaterally. Contralateral recordings were used for quantitative measures of eFFR. Waveforms from individual trials were saved to computer disk, and averages across trials were displayed online. Offline analysis and illustration of recorded waveforms employed zero-phase bandpass filters having passbands of 300 to 1500 Hz for the eABR and 50 to 1000 Hz for the eFFR; the filter order was set at 1, which was doubled by the bandpass design and doubled again by use of the MATLAB *filtfilt* function, resulting in fourth-order filters.

The stimuli for eABRs were single pulses, 10 per second. Current levels, including a zero-current condition, were varied pseudo-randomly among pulses so that each level was tested once in a random order, then each was presented again in a different random order, and so on until 100 or 200 repetitions of each level were tested. An electromyographic (EMG) signal, due to activation of the facial nerve, often was recorded at high stimulus level, more often for CI than for IN stimulation. The presence of the EMG at high stimulus levels limited the range of stimulus levels that could be tested.

The eFFR was measured with trains of pulses, 500 ms in duration, fixed in level, and varied in pulse rate among trials; pulse trains were presented at 1200-ms intervals from onset to onset. The stimulus level in each case was selected to be high in the dynamic range of scalp responses but at least 2 dB lower than the threshold for the EMG; levels of eFFR stimuli are discussed in the “Results” section. Pulse rates varied from 43 through 643 pps in step sizes ranging from 5 to 32 pps. The pulse rates were selected to provide the desired sampling of the eFFR transfer function with the restrictions that rates were integer divisors of the output sample rate and avoided the 60-Hz powerline frequency or its harmonics. All the pulse rates were tested in pseudo-random orders until 40 repetitions of each rate were tested, including 20 repetitions each of cathodic- and anodic-leading polarities.

### Data Analysis

Scalp recordings were contaminated with electrical artifacts from the stimulus pulses. This contamination was mitigated partially by use of cathodic- and anodic-leading pulse polarities that alternated between trials, which when averaged tended to cancel the artifact while largely preserving the biological waveforms. Additional artifact reduction for the eFFR waveforms employed template subtraction, blanking, and interpolation, as described by [11]. Briefly, a template of the artifact for each polarity was computed by averaging across all pulse rates within the interval of 0–1400 µs after onset of each pulse train. That interval captured the response to the first pulse in each train, which occurred 700 ms after the offset of the preceding pulse train and was unaffected by the rate of the ensuing pulses in each train. The template then was aligned in time to the individual pulses of the waveform for each pulse rate and polarity, which yielded a template sequence that was subtracted from the waveform. After template subtraction, a segment of each waveform, from 50 to 850 µs after each pulse onset, was blanked, and a line was interpolated from the beginning to the end of the blanked period. After template subtraction, blanking, and linear interpolation of traces from each polarity, cathodic- and anodic-leading waveforms were averaged together. Artifact reduction for the eABR waveforms, which were elicited by single electrical pulses, required only the linear interpolation and polarity averaging steps.

Thresholds for eliciting eABRs were computed using procedures from signal detection theory [15, 16]. Given 100 or 200 trials at each stimulus level, 400 bootstrap averages of 100 or 200 trials were drawn with replacement from all the trials. For each level, an empirical receiver operating characteristic (ROC) curve was formed from amplitudes of responses to a given stimulus and from the next higher level; the amplitudes were root-mean squares in the time range from 1 to 4 ms after pulse onset. The area under the ROC curve gave the probability that the response to the higher level was larger than that to the lower level. That probability was expressed as a *z*-score and multiplied by √2 to obtain the detection index, *d’*. The cumulative sum of *d’* values was formed from the lowest to the highest stimulus levels and was interpolated in 0.1-dB steps. The eABR threshold was given by the lowest interpolated stimulus level at which the cumulative *d’* was ≥ 1.

Recordings of the eFFR were analyzed as the transfer functions of phase-locked responses measured at the scalp as a function of pulse rate. For each pulse rate, Fourier analysis of the eFFR waveform yielded the amplitude and phase at the fundamental frequency. The response at each pulse rate was tested for statistically significant phase locking by the Rayleigh test for non-uniformity of trial-by-trial phase values [17]. Amplitude and phase transfer functions of the eFFR were interpreted as sums of putative cochlear nucleus, higher brainstem, midbrain, and thalamocortical/cortical generators, each synchronized to the stimulus pulse rate [11, 18–20]. The phase transfer function in each case was unwrapped so that the phase lags relative to electric pulses increased monotonically with increasing pulse rate; phase steps of 2π radians were added as needed to prevent differences of greater than ± π radians between phase values at adjacent pulse rates. The unwrapped cumulative phase transfer functions of eFFRs were fit initially with five line segments using piecewise linear regression, then adjacent segments whose slopes did not differ significantly were combined, yielding a total of two to five segments in each phase transfer function. The slopes of each line segment divided by $2\pi$ yielded a group delay, each of which was interpreted as the delay from the stimulus pulse to the neural generator that dominated the eFFR across the corresponding range of stimulus pulse rates. The highest pulse rate within each segment was taken as the maximum rate at which stimulus-synchronized activity of the corresponding generator dominated the phase of the scalp-recorded response.

A major interest of this study was to test whether a key result from our previous short-term single- and multi-unit recordings from the ICC could be replicated with long-term implantation and scalp recordings. That result was that transmission of TFS from the electrically stimulated auditory nerve to the midbrain was enhanced by IN stimulation of apical fibers compared to CI stimulation of higher-CF fibers [10]. For that reason, much of our eFFR analysis here focused on latencies and pulse rates relevant to ICC responses. Specifically, latencies of neural spikes in the ICC in our acute invasive study were ≥ 4 ms, and pulse rates above or below 300 pps tended to differentiate ICC synchrony to IN compared to CI stimulation.

## Results

### Electrically Evoked Auditory Brainstem Response (eABR)

**Fig. 2:**
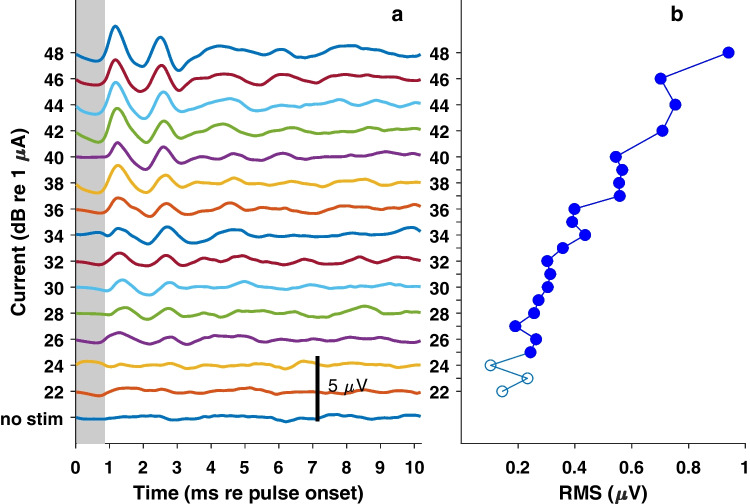
eABR waveforms recorded from one cat, ipsilaterally to the stimulated ear, passband 300 to 1500 Hz. Stimuli were single biphasic pulses, 82 µs/phase. **a** The colored curves show the waveforms at current levels in 2-dB increments. *no stim* indicates background activity in the absence of overt stimulation. The vertical gray bar indicates the 50–850 µs time range corresponding to blanking and linear interpretation for electrical artifact rejection. **b** RMS voltages recorded during 1 to 4 ms after the pulse onset in 1- or 2-dB increments of stimulus current; current is given on the vertical axis, sharing the scale with Fig. 2a. Filled symbols denote recordings that were classified as above threshold according to the signal detection procedure described in the Methods. Cat AP, post-implantation day 78

Measures of the eABR quantified the sensitivity of the auditory brainstem to single electrical pulses and the growth of response magnitude with increasing stimulus current. Measures of the eABR threshold at 2–3-week intervals assessed the stability of stimulation over the course of months after deafening and implantation. Figure 2a shows eABR waveforms from one cat recorded in response to single pulses from an IN electrode, 78 days post-implantation, in 2-dB steps of current; the background activity in a no-stimulus condition is also shown. The vertical gray bar indicates the 50–850-µs time range corresponding to blanking and linear interpretation for electrical artifact rejection (described in the “Methods” section). We attribute the first positive-going deflection, at peak latency ~ 1.2 ms, to the cochlear nucleus; the earlier compound action potential from the auditory nerve would have been lost in the artifact-rejection procedure. Waveform morphology varied among animals, recording laterality, and filter settings.

Estimates of eABR thresholds were based on the root-mean-squared (RMS) voltage in the passband from 300 to 1500 Hz in the time window from 1 to 4 ms after pulse onset. Figure 2b plots the RMS values corresponding to the waveforms in Fig. 2a; stimulus current levels are given here on the vertical axis in 1-or-2-dB increments (aligned with the traces in Fig. 2a), and amplitude is given on a linear voltage scale on the horizontal axis. As detailed in the “Methods” section, we computed the cumulative *d’* across successive current levels beginning with the no-stimulus condition. The filled symbols in Fig. 2b denote RMS values at which the cumulative *d’* was ≥ 1, i.e., above threshold.

**Fig. 3:**
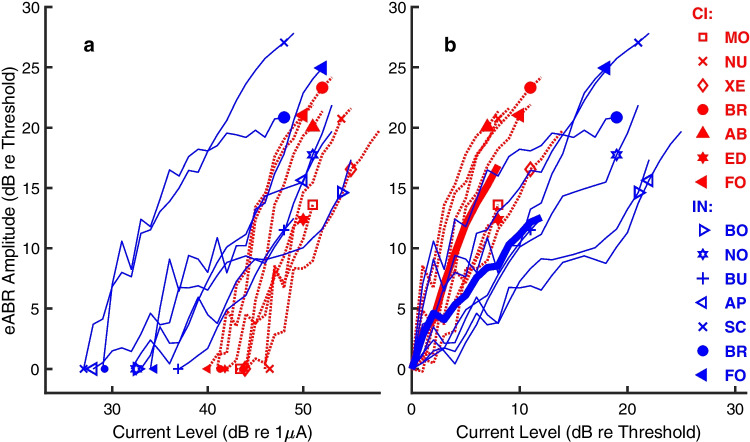
Growth of eABR amplitude (vertical axis) with increasing level (horizontal axis) of 82 µs/ph biphasic pulses. eABR amplitude is expressed as dB re the eABR amplitude at threshold. Each curve represents data from one animal studied ~ 70 days after implantation, blue for IN stimulation and red for CI stimulation. Symbols assigned to each cat (defined in the key) are maintained in this and in Figs. 4, 5, 6, 10, and 11. Symbols near the base of each curve indicate the eABR amplitude at threshold for each animal, and symbols near the top of each curve indicate the eABR amplitude at the stimulus level that was used for eFFR measurements. **a** eABR amplitudes shown at current levels expressed as dB re 1 µA. **b** Amplitudes shown at current levels expressed as dB re the threshold for each curve. The thick lines indicate the means across animals for IN and CI stimulation; the means shown by the thick lines were computed only across the range of suprathreshold levels that were tested in all the CI or all the IN animals

The growth of RMS of eABR amplitudes with increasing stimulus current is shown in Fig. 3a for each of the cats; data from test sessions ~ 70 days after implantation are shown for IN (blue) and CI (red) stimulation. Here, eABR amplitudes are given on the vertical axis as dB re the eABR amplitude at threshold for each cat, and current is given on the horizontal axis as dB re 1 µA. The symbol near the base of each curve (at 0 dB) indicates the eABR amplitude at threshold for that animal; the symbol near the top of each curve is explained in the following section. The maximum amplitudes of recorded eABRs were around 20–25 dB above the amplitude at threshold for both IN and CI stimulation. Note that the maximum amplitude that could be recorded for CI and, sometimes, for IN stimulation was practically limited by the myogenic (EMG) response that often was encountered at the highest stimulus levels. Despite the similar ranges of recorded amplitudes, the growth plots for IN and CI stimulation differed markedly in the stimulus ranges that elicited those response ranges. The thresholds for IN stimulation, expressed re 1 µA, are all lower than those for CI stimulation in the illustrated cases. Nevertheless, the amplitudes for both types of stimulation grow to maxima at around the same highest tested stimulus levels, around 50 dB re 1 µA. Consequently, the slopes of growth plots are shallower for IN than for CI stimulation. The difference in slopes is more evident in Fig. 3b, in which stimulus currents are expressed relative to the threshold for each plot. The thick lines plot the mean growth functions for IN (blue) and CI (red) stimulation. Slopes were shallower for IN than for CI stimulation.

**Fig. 4:**
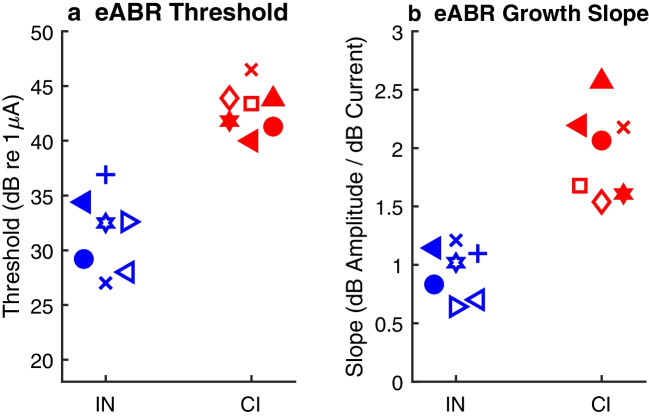
eABR level sensitivity for intraneural (IN) and cochlear-implant (CI) stimulation at ~ 70 days post implantation. **a** Thresholds. **b** Slopes of eABR growth quantified as eABR amplitude in dB/stimulus level in dB. In both panels, symbols are jittered in the horizontal dimension to aid in visualization. The assignment of symbols to cat codes is as in Fig. 3

The distributions of eABR thresholds are shown in Fig. 4a. Means were 31.5 dB re 1 µA for IN stimulation and 43.0 dB for CI stimulation (two-sample two-tailed *t*-test: *t* = 7.2, *p* = 1.0 × 10^−5^, *df* = 12). Slopes are shown in Fig. 4b as dB amplitude/dB current. Mean slopes were 0.95 for IN and 2.0 for CI stimulation (*t* = 6.2, *p* = 4.9 × 10^−5^, *df* = 12). The relevance of growth-of-amplitude slopes to tonotopic spread of excitation is considered in the “Discussion” section.

**Fig. 5:**
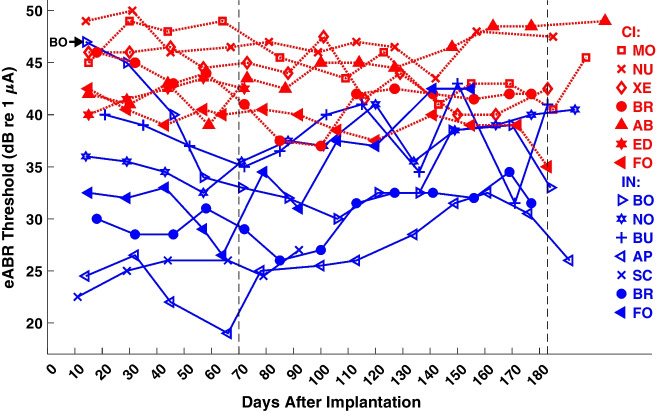
eABR thresholds versus days after implantation. Each symbol type indicates data from one animal keyed in the column at the right. Symbols are jittered by ± 1 day when necessary to avoid overlap. Colors and line styles indicate CI (red, dotted lines) and IN (blue, solid lines) stimulation. Filled markers indicate CI + 1 cats that were implanted with both CI and IN devices in the same ear in the same operation; CI and IN electrodes in those animals were tested in separate series of trials. The black arrow labeled BO (upper left) indicates the beginning of data for Cat BO for which the first three measurements were deemed invalid; those were the first test sessions in which such low thresholds were encountered, and the tested level range did not extend to sub-threshold levels. The vertical line at day 70 indicates the day closest to the test days that were used for two-sample two-tailed *t*-tests mentioned in the main text. The vertical line at day 183 indicates the target study longevity of 6 months

The eABR thresholds for the various cats are shown as a function of days after implantation for IN (blue) and CI (red) stimulation (Fig. 5); filled markers indicate the animals that were implanted with CI + 1 devices. Two characteristics are most prominent. First, the distributions of IN and CI thresholds are largely non-overlapping, with CI thresholds higher than IN thresholds. The threshold difference was shown for single test sessions in Fig. 3a, but Fig. 5 shows that the difference persisted across months after implantation. Comparison of CI thresholds between CI-only and CI + 1 cases showed no consistent elevation of threshold in the CI + 1 cases, which alleviates a possible concern that the passage of IN electrodes through the fibers of the basal turn might have been injurious to those basal-turn fibers; the basal-turn fibers are the fibers activated by CI electrodes in the present study. Throughout this report, we assessed summary data on the test day for each animal closest to 70 days after implantation. That time point was chosen as a reasonably long period of chronic implantation that was achieved by all the cats in the study, even those that had hardware failures at later times.

The second prominent characteristic of the data in Fig. 5 was that thresholds for individual animals showed little or no systematic variation across as long as 6 months (183 days) after implantation, even though those deafened animals received no stimulation during this time other than during the recording sessions, ~ 1–2 h every 2–3 weeks. Thresholds from the first three test days from cat BO were systematically overestimated and are illustrated but were excluded from across-day quantitative analysis. Among the seven tested IN electrodes, six reached our target longevity; we include Cats FO and BR among the 183-day count although connectors on those cats failed a few days short of the target, after days 155 (FO) or 177 (BR). Cat SC did not reach the 6-month mark because the skull-mounted connector became fouled, leading to marked increases in impedances after day 92. A two-way analysis of variance (ANOVA) for all the illustrated data (omitting the first three measurements from BO) showed a significant main effect of IN-vs-CI stimulation (*F* (1, 139) = 217, *p* < 10^−15^) but no significant effect of days after implantation in 14 blocks of 14 days (*F* (13, 139) = 1.1, *p* = 0.36). We take the main effect of stimulation type as indication that IN stimulation activated the auditory pathway at lower currents than did CI stimulation. The absence of significant effect of days after implantation indicates that there is no evidence for an effect of implantation on the functional health of the auditory nerves in these animals.

### Electrically Evoked Frequency Following Response (eFFR)

**Fig. 6:**
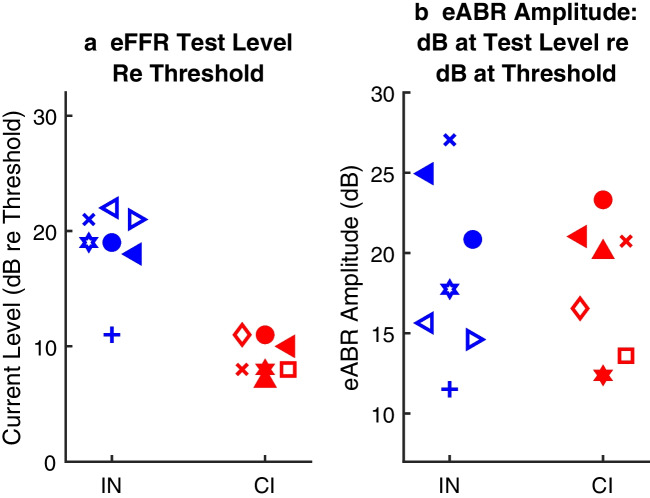
Stimulus levels used for eFFR measurements. **a** eFFR stimulus level re the single-pulse eABR threshold for each animal. **b** eABR amplitudes measured at the eFFR stimulus levels, given as dB re the amplitude of the eABR at threshold for each animal. Symbols are jittered in the horizontal dimension to aid in visualization. The assignment of symbols to cat codes is as in Fig. 3

The eFFR provided a measure of transmission of TFS from the auditory-nerve stimulus to various brainstem, midbrain, and thalamocortical generators. We tested stimulus levels that were rather high in the dynamic ranges of eABR growth functions; the levels that were used for eFFR testing are marked by the symbols near the tops of the eABR growth plots in Fig. 3. The eFFR test levels were always set at least 2 dB below levels that elicited electromyogenic signals from facial nerve activation, when evident. Given the shallower slopes of IN eABR growth functions, that means that the highest practical stimulus levels were higher above threshold for IN than for CI stimulation. That is shown in Fig. 6a, which plots the distributions of levels that were tested, expressed as dB re threshold. Mean test levels re threshold were 19 dB for IN and 9.0 dB for CI stimulation (*t* = 6.4, *p* = 3.5 × 10^−5^, *df* = 12). Figure 6b plots the eABR amplitudes at the eFFR stimulus levels. Expressed in that way, the distributions of test levels for IN and CI overlap extensively, largely due to the shallower growth functions for IN stimulation. Mean amplitudes were 19 (IN) and 18 (CI) dB re threshold (*t* = 0.25, *p* = 0.80, *df* = 12). That result indicates that the stimulus levels that were chosen for eFFR testing were similar between IN and CI stimulation with respect to eABR amplitudes expressed relative to amplitudes at threshold.

**Fig. 7:**
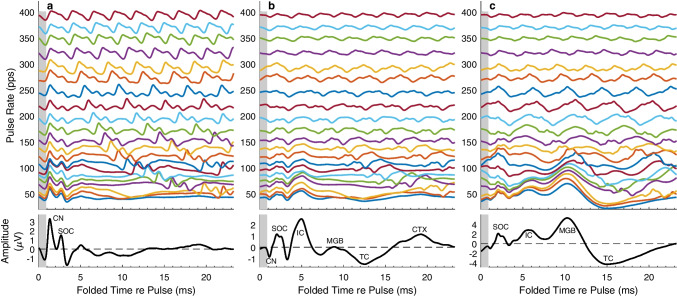
Folded eFFR waveforms, passband 50–1000 Hz. Waveforms recorded from two IN cats were elicited from 50 to 500 ms after the onsets of electric pulse trains, with the pulse rates indicated on the vertical axes. The time base for each pulse rate is folded on the period of that rate times the smallest integer multiple that totaled ≥ 23 ms. The vertical gray bar in each panel shows the time range (from 50 to 850 µs) that was blanked and interpolated for artifact rejection; for clarity of presentation, the gray bar is shown only for the first pulse in each trace, although artifact rejection was applied to every pulse. The bottom trace in each panel, in black, shows the response to the lowest pulse rate (43 pps) expanded in the amplitude dimension. **a** Cat AP (78 days after implantation); ipsilateral recording. **b** Cat AP (day 78); contralateral recording. **c** Cat SC (day 79); contralateral recording. *CN*, cochlear nucleus; *SOC*, superior olivary complex; *IC*, inferior colliculus; *MGB*, medial geniculate body; *TC*, thalamocortical projection; *CTX*, primary auditory cortex

Examples of time-folded eFFR waveforms from three series of responses to IN stimulation are given in Fig. 7. These waveforms were derived from samples at 50–500 ms re the onset of each pulse train; the response to the first 50 ms of each train was excluded to eliminate the first pulse, which tended to elicit a strong response irrespective of the rates of ensuing pulses. Each illustrated waveform is an average that is folded on a time range equal to the period of the pulse rate multiplied by the smallest integer multiple of that period that was ≥ 23.3 ms; 23.3 ms was the stimulus period of the lowest tested rate (43 pps). That means that the waveform for, say, the 152.6-pps pulse rate was folded on its period, 6.55 ms, times 4, which gave a total fold time of 26.2 ms. That folded waveform then was truncated to 23.3 ms, the length of the illustrated time axis. The colored waveforms represent pulse rates increasing from bottom to top; for clarity of presentation, the waveforms are shown only for rates < 400 pps. The waveform in black at the bottom of each column is the response to the 43-pps pulse rate, enlarged in amplitude to aid visibility of waveform features. The same features are present in all three cases but differ in prominence among recording-electrode placement and animals. The vertical gray bar in each panel shows the time range (from 50 to 850 µs) that was blanked and interpolated for artifact rejection; for clarity of presentation, the gray bar is shown only for the first pulse in each trace, although artifact rejection was applied to every pulse.

The waveforms in Fig. 7a were ipsilateral recordings from cat AP. The enlarged (black) trace for the 43-pps rate shows a prominent vertex-positive deflection at ~ 1.30 ms, which, based on the latency, we attribute to the cochlear nucleus (CN); the compound action potential from the auditory nerve at latency < 1 ms was lost in the artifact-reduction procedure. The CN deflection was followed by another positive-going deflection, at ~ 2.7 ms, and a negative-going deflection at ~ 3.5 ms, which we attribute to the superior olivary complex and possibly additional brainstem generators (denoted here collectively as SOC). The CN deflection can be seen at all the illustrated pulse rates (shown in colors). At the higher rates, the CN deflection increases slightly in latency, from 1.33 to 1.48 ms, and broadens, both of which we attribute to habituation at the high pulse rates. There is a positive deflection at latency ~ 5 ms, which is within the range of latencies of electrically evoked spike activity in the ICC [10, 21]. The waveforms in Fig. 7b are the contralateral recordings from the same cat (AP) as in Fig. 7a, c shows contralateral waveforms from cat SC. Viewed from the contralateral side, the CN deflection is negative-going and smaller than in the ipsilateral recording, but the IC deflection at ~ 5 ms is especially prominent, particularly in Fig. 7b. The IC deflection in this laterality is evident in the colored waveforms at pulse rates up to ~ 200 pps or higher. There is a broad positive deflection centered at ~ 9–10 ms at the lowest pulse rate, more prominent in Fig. 7c. That latency is consistent with activity in the medial geniculate body (MGB; [22]).

Figure 7b, c each show two deflections that likely originated in the auditory cortex. The first is a negative deflection (labeled TC) centered at 12 ms (in Fig. 7b) or 15 ms (Fig. 7c), which likely reflects activity in thalamic input layers of primary auditory cortex [23]. That first deflection likely also includes the thalamocortical excitatory post-synaptic potentials. The second deflection (labeled CTX) is positive at 19 ms in Fig. 4b and > 23 ms in Fig. 4c, similar to group delays of spike activity in the unanesthetized primary auditory cortex of cats in response to acoustic click trains ([24] and unpublished data from that study). The sizable delay between the thalamic input and group delays of spike activity agrees with the intra-cortical temporal filter delay of 7.5 to ~ 14 ms between first-spike latencies and group delays described by Eggermont for cat acoustic click trains [25]; similar delays between cortical first-spike latencies and group delays were reported for CI stimulation in guinea pigs [26]. The colored lines in Fig. 7c show the MGB deflection synchronizing to pulse rates > 108 pps, whereas the TC deflection is evident only to about 55 pps.

At the low pulse rate shown in black in Fig. 7, one can easily identify waveform deflections arising from putative generators. At higher pulse rates, however, long-latency deflections are overtaken by short-latency responses to following pulses. This is particularly evident for the CTX deflection in Fig. 7b, and the MGB and TC deflections in Fig. 7c. The overlap between long-latency responses to earlier pulses and short-latency responses to later pulses means that the eFFR recorded at rates higher than ~ 50 pps is given by the sums of multiple brainstem, midbrain, and thalamocortical generators.

**Fig. 8:**
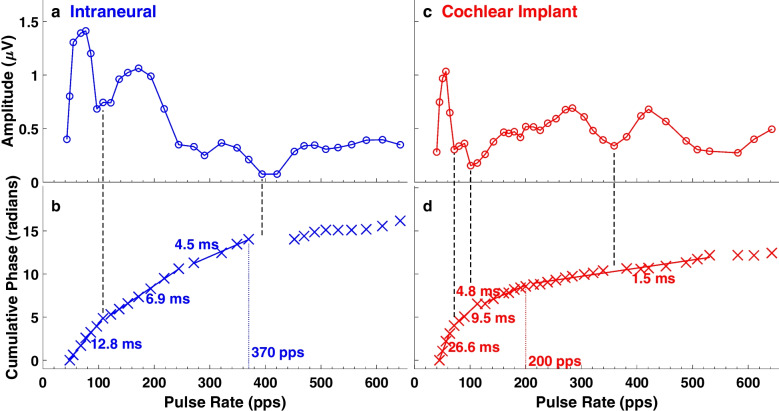
Pulse-rate transfer functions: amplitudes (**a**, **c**) and cumulative phases (**b**, **d**) are the Fourier amplitudes and phases at the component closest to each pulse rate. The vertical dashed lines passing from **a** to **b** and from **c** to **d** indicate nulls in amplitude at which phase was ambiguous or the rate of growth of phase lag declined. The cumulative phases each are fit with three or four line segments using piecewise linear regression. Group delays, indicated in ms, were computed from the slopes of the line segments. The right-most end of each line segment indicates the pulse-rate cutoff, which was the maximum pulse rate at which the eFFR was dominated by a generator having the corresponding group delay. In these examples, the pulse-rate cutoff for a putative midbrain generator having a latency of ≥ 4 ms is indicated by the dotted vertical lines at 370 pps (**a**) and 200 pps (**b**). **a**, **b**: Cat BO day 57; **c**, **d**: Cat XE day 73

The sum-of-generator concept has been explored in normal hearing (cat: [18]; rabbit: [19]; human: [20]) and CI hearing (cat: [11]). Here, the sum-of-generators characteristic of eFFR recordings was evident in the transfer functions from stimulus pulse rates to eFFR recordings. Amplitude transfer functions from IN (Fig. 8a) and CI (Fig. 8c) stimulation cases exhibited prominent peaks and dips, which are characteristic of a sinusoid added to itself with multiple delays. Within particular CI or IN electrodes in individual cats, the magnitudes of peaks and dips could vary considerably across test sessions. Nevertheless, the pulse rates corresponding to peaks and dips in amplitude transfer functions were remarkably consistent across various test sessions within each cat. For example, we identified the pulse rates at three dips in the amplitude transfer function in the ~ 70-day test session for each cat and compared those rates with corresponding rates in the ~ 100-day test session. We scored a “hit” whenever the rates at 70 and 100 days differed by no more than 32 pps, which was the smallest pulse-rate increment that was tested across all ranges of pulse rates. Hits were observed in 39 (92.9%) of 42 cases (i.e., three dips in each of seven CI cases and seven IN cases = 42); the median magnitude of error across hits and misses was 0 pps (interquartile range 0 to 15.5 pps). The few misses were instances in which dips were very shallow in one or both of the test sessions. The variation in transfer functions was considerably greater across the various cats. We evaluated the inter-cat differences by testing the same 42 conditions (three dips × 14 cases) with 200 random permutations of the day-70 day sessions from one cat and the day-100 sessions from a different cat. That yielded substantially lower hit rates: median = 61.9%, 95th percentile = 71.4%.

The phase transfer function from the same cases as Fig. 8a, c are shown in Fig. 8, d. Vertical dashed lines between upper and lower panels show instances in which phase values were poorly defined at pulse rates corresponding to nulls in the amplitude transfer function. Phase transfer functions typically showed multiple segments of relatively straight increasing phase lag with increasing pulse rate. In the example in Fig. 8b, from an IN case, piecewise linear regression could fit most of the phase values with three line segments, running from pulse rates of 48 to 108 pps, 122 to 244 pps, and 271 to 370. The slope of each of those segments divided by $2\pi$ yielded the corresponding group delay, equal to 12.8, 6.9, and 4.5 ms in this example. We assume that the group delays reflect the neural latencies of particular generators in the auditory pathway. The group delays here are consistent with scalp recordings dominated by thalamocortical (12.8 ms), midbrain to thalamic (6.9 ms), and IC (4.5 ms) generators. In this example, 370 pps, marked with a blue dotted line, was taken as the cutoff rate for synchrony dominated by midbrain levels of the auditory pathway. In the example from a CI case (Fig. 8b, d), the phase transfer function could be fit well with four line segments. The segment having a group delay consistent with a midbrain generator, 4.8 ms, only reached to 200 pps (red dotted line).

**Fig. 9:**
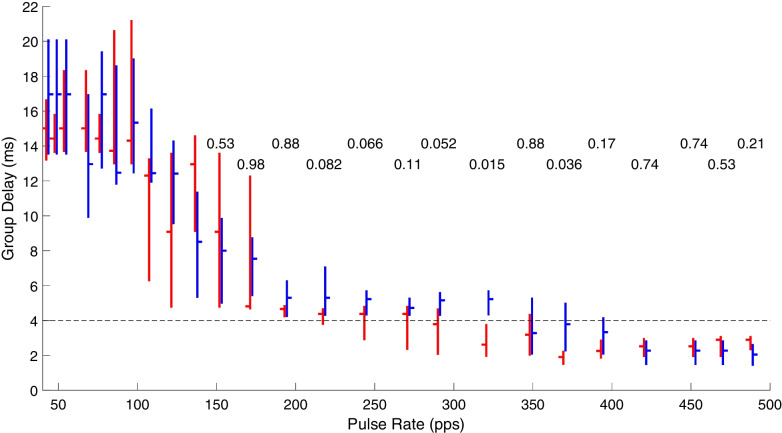
Distributions of group delays as a function of stimulus pulse rate. Data are from the test days closest to 70 days after implantation compiled across cats. The analysis included seven IN cases and seven CI cases, but group delays were not measurable at all pulse rates for all cats. For that reason, there are four to seven (median = 6) cats included for each IN condition and two to seven (median = 6) for the CI conditions. Each pair of red (for CI stimulation) and blue (for IN) vertical lines represents the interquartile range of group delays computed for each pulse rate, and the short horizontal lines indicated the median values. The values printed across the top are *p* values for two-sample *t*-tests at each pulse rate; there was no assumption that the CI and IN distributions had equal variance. The *p* values at rates < 150 pps are omitted for clarity, but all were > 0.14

Based on the piecewise linear regression analysis of phase transfer functions, we compiled across animals the group delays of the generators that dominated the response to each pulse rate. The distributions of those group delay values are shown in Fig. 9 for seven IN cases (in blue) and seven CI cases (in red). Interquartile ranges (vertical bars) and medians (short horizontal bars) are given for each pulse rate and IN and CI device. We limited this analysis to pulse rates < 500 pps because the eFFR at higher rates was dominated by short-latency brainstem activity in which group delays were poorly defined in the piecewise regression analysis. The horizontal rows of numbers in the figure gives the *p* values for two-sample *t*-tests comparing the group delays for CI-versus-IN stimulation at each pulse rate; *p* values at rates < 150 pps were all > 0.14 but are omitted for clarity. The *t*-tests showed significant differences at rates of 321 and 370 pps, but none of the *p* values was significant after Bonferroni correction for 19 repeated observations.

A two-way ANOVA of the data in Fig. 9 across the 19 pulse rates from 68 to 421 pps showed a main effect of pulse rate (*F* (18, 174) = 24, *p* < 10^−15^) but no significant effect of CI-versus-IN stimulation (*F* (1, 174) = 0.43, *p* = 0.51). The main effect of longer group delays at lower pulse rates is expected because midbrain and forebrain auditory structures having long latencies (and long group delays) are known to synchronize only to relatively low rates [10, 24, 27, 28]. The absence of a significant main effect of CI-versus-IN stimulation seemingly is contrary to our expectation of enhanced brainstem synchrony to IN stimulation, which is based on invasive ICC unit recordings [10]. The present analysis, however, includes a range of high pulse rates in which eFFR amplitudes were low and for which the response is dominated by peripheral sites for which the group delays would not be expected to differ between stimulus type. Also, there was a range of low rates, ≤ 150 pps, for which synchrony of thalamocortical generators would be expected to show a ceiling for either electrode type. The figure shows, however, that group delays consistently are higher for IN than for CI stimulation across pulse rates around 300 pps and group delays > 4 ms, which are relevant to synchronized unit activity in the ICC [10]. Nearly all the units recorded in the previous ICC study showed first-spike latencies and group delays from 4 to 9 ms, and the difference in ICC synchrony between CI and IN stimulation became conspicuous at pulse rates around 300 pps and higher. For those reasons, we define eFFR group delays ≥ 4 ms as a region of interest in the present study and henceforth will use the *midbrain sync cutoff* to refer to the highest stimulus pulse rate at which the group delay was ≥ 4 ms. In Fig. 9, the eight pulse rates in which the interquartile range for IN and/or CI stimulation included 4 ms ranged from 218 to 394 pps. If we limit our two-way ANOVA to that range of rates, we observe significant main effects of pulse rates (*F* (7, 76) = 4.6, *p* = 0.00024) and stimulation type (*F* (1, 76) = 23, *p* = 7.8 × 10^−6^). The main effect of stimulation type across that limited range of pulse rates accords with the previous result that substantially more ICC neurons synchronized at pulse rates of ≥ 300 to IN compared to CI stimulation [10].

**Fig. 10:**
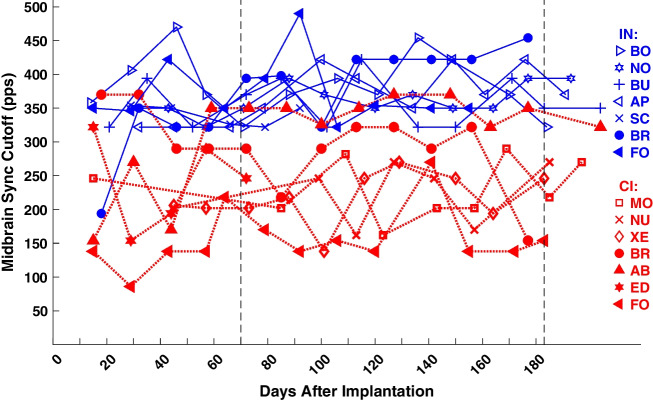
Midbrain sync cutoffs as a function of days after implantation. The midbrain sync cutoff is the highest pulse rate at which the analysis of the eFFR phase transfer functions showed a group delay ≥ 4 ms. Markers are jittered by ± 1 day when necessary to avoid overlap. Filled markers denote CI + 1 implants. Blue color and solid lines denote IN stimulation; red color and dotted lines denote CI stimulation. Other details as in Fig. 5

The data in Fig. 9 were collected from the single test session for each cat that was nearest to 70 days after implantation. For the goals of the study, it was of interest to monitor possible long-term changes in midbrain sync cutoffs. Figure 10 shows midbrain sync cutoffs as a function of days after implantation for IN (blue) and CI (red) stimulation. With few exceptions, eFFR at group delays consistent with midbrain generators (i.e., ≥ 4 ms) was present at higher pulse rates for IN than for CI stimulation. The trends across days made a few large swings, which we attribute to difficulty in interpreting phase spectra around dips in amplitude spectra and, in some cases, difficulty in placing electrodes to record a dominant midbrain generator. Nevertheless, there were no consistent long-term trends across post-implantation time. A two-way ANOVA of eFFR cutoff showed a significant main effect of IN-versus-CI stimulation type (*F* (1, 115) = 114, *p* < 10^−15^; means across all days, 367 pps for IN and 241 pps for CI), whereas there was no significant main effect of days after implantation compiled in 14 groups of 14 days (*F* (13, 115) = 0.87, *p* = 0.59); similar significance of device type (*F* (1, 106) = 94, *p* < 10^−15^) and lack of significance of days after implantation (*F* (13, 106) = 0.84, *p* = 0.62) were observed when the analysis was repeated after excluding the test days that were used in Fig. 9. We take the main effect of device type, that midbrain synchrony cutoffs were higher for IN than for CI stimulation, as indication that IN stimulation activated brainstem pathways that transmit TFS more effectively than do pathways activated by CI stimulation. As in the case of stable eABR thresholds, the lack of significant effect of days after implantation fails to support any hypothesis of an ill effect of implantation and stimulation on the maintenance of auditory-nerve function.

### Temporal Acuity Maintained After Deafening

**Fig. 11:**
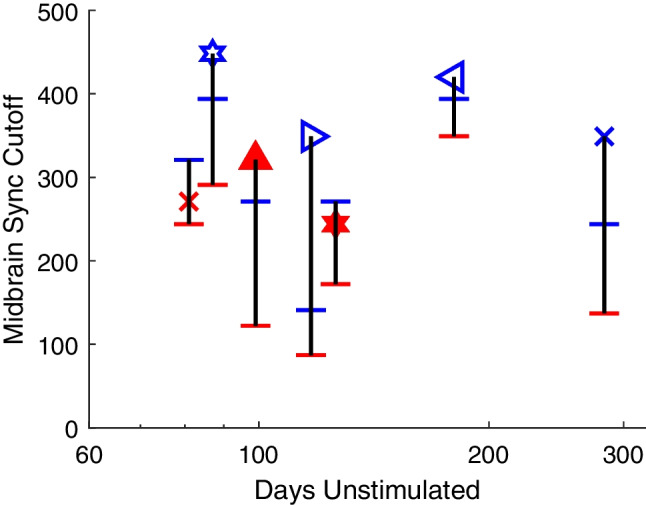
Loss of temporal acuity after > 80 days of no stimulation. Each vertical triad of symbols represents data from one animal, with the horizontal dimension plotting the stated number of days after the cessation of routine electrical stimulation. The symbols, corresponding to the cat codes defined in Fig. 3, indicate the midbrain synchrony cutoff obtained with the chronic electrode in the last routine recording session, either IN (blue) or CI (red), and the horizontal lines indicate the values obtained in a terminal experiment with acute implantation of IN (blue) and CI (red) electrodes in the formerly un-implanted ear

In a previous study, we found that cats that were deafened and left unstimulated for ~ 6 months showed a marked degradation of temporal acuity, as measured by loss of phase-locking of single- and multi-unit activity in the ICC [13]. Animals in the present study were similarly deaf for ~ 6 months but were stimulated unilaterally through an implanted electrode for ~ 1–2 h at 2–3-week intervals. Those animals showed no systematic loss of temporal acuity as measured by the eFFR. In a subset of those animals, we tested temporal acuity in terminal experiments more than 80 days after cessation of routine stimulation. Those animals were sedated, and CI and IN electrodes were implanted acutely, one at a time, in the formerly un-implanted ear. Figure 11 shows the eFFR midbrain synchrony cutoffs from those seven cats. The red or blue symbol indicates the value that was recorded with the chronic stimulating electrode in the last routine recording session; each of these animals had only one or the other type of chronic electrode. The red and blue horizontal lines indicate values obtained in the terminal recording session with the acutely implanted electrodes. In every case, the acute IN stimulation yielded midbrain synchrony at higher rates than did acute CI stimulation (pairwise *t* (6) = 6.8, *p* = 0.00051). This replicates the effect of device on midbrain synchrony, observed in the analysis of the data presented in Figs. 9 and 10, with a fresh data set. The maximum synchrony rates from the acute CI and IN stimuli after > 80 days of no stimulation generally were lower compared to the chronic stimulation in those animals (pairwise *t* (6) = 3.4, *p* = 0.014). In the two exceptions, at 81 and 126 days unstimulated, the acute CI value was lower than the chronic CI value, although the acute IN value was higher than both CI values. The results of these terminal experiments support the view that temporal acuity was sustained in deafened animals by routine electrical stimulation and that acuity was degraded after a period of > 80 days of no stimulation.

## Discussion

We have shown previously that an IN electrode inserted into the trunk of the auditory nerve can selectively excite low-frequency fibers originating from the cochlear apex [8] and that those fibers activate brainstem pathways that are specialized for high temporal acuity [10]. Apical fibers and their corresponding brainstem pathways cannot be stimulated selectively by today’s cochlear implants, both because most implants lie in the basal and middle turns and because selective stimulation in the apical turn is precluded by the geometry of the apical spiral ganglion (discussed below). We have argued that the failure to activate apical pathways selectively might account for the poor sensitivity to TFS exhibited by CI users. That temporal insensitivity could, in turn, account for CI users’ impaired use of pitch contours and interaural timing cues for understanding speech from one talker amid other competing sounds. Here, we evaluated the feasibility of long-term IN stimulation with an electrode implanted chronically in the cat auditory nerve. The results show that IN stimulation yields the anticipated improved temporal acuity: eFFR at midbrain latencies was evident at higher pulse rates for IN than for CI stimulation. Moreover, measures of thresholds for brainstem activation and of temporal acuity showed no degradation of nerve function for as long as 6 months after implantation. Here, we make a few quantitative comparisons between the present chronic measures and those from our previous acute studies. Then, we discuss: (1) some design considerations and results from the iridium-oxide IN electrode; (2) evidence that stimulation at intervals as infrequent as 2–3 weeks can maintain temporal acuity in the auditory pathway; (3) challenges of selective apical stimulation with intrascalar electrical or optical devices; and (4) prospects of the CI + 1 device for enhancing prosthetic hearing.

### Comparison with Short-Term Invasive Recordings

In our previous short-term (< 24 h) experiments in cats, we stimulated with single pulses from IN or CI electrode arrays and recorded extracellular spikes at 32 sites along the tonotopic axis of the ICC [8]. The tonotopic spread of activation was markedly more restricted for IN stimulation: about 1/3 of that for CI stimulation. Thresholds for IN stimulation averaged ~ 25 dB lower for IN than for CI stimulation. Dynamic ranges were broader: > 13 dB for IN stimulation compared with ~ 3 dB for CI stimulation.

In the present study, eABR thresholds showed a mean IN-to-CI difference of only ~ 13 dB, which we regard as substantial but appreciably smaller than that observed in the acute experiments. The main difference between the two studies is that, here, IN stimulation gave eABR thresholds about 10 dB higher than the thresholds for ICC spike thresholds in the 2007 study [8], whereas CI stimulation gave eABR thresholds about 2.3 lower than for ICC spikes. Those differences might be due to the sizes of stimulated nerve fiber populations needed to activate neurons in direct contact with a microelectrode in the ICC compared to the much-larger activated population needed to elicit a far-field eABR at the scalp. If the spread of excitation is more restricted from IN than from CI stimulation [8], it might be that activation of brainstem populations large enough to elicit scalp-recorded eABRs required levels that were higher in the IN dynamic range than was the case for CI stimulation.

As in the previous study, dynamic ranges here were substantially broader for IN than for CI stimulation. Slopes for growth of eABR amplitudes were shallower by about half for IN compared to CI stimulation, and the dynamic range of stimuli eliciting threshold to maximal amplitudes (below EMG levels) was more than twice as broad for IN compared to CI stimulation. The present experimental design, with scalp-recorded eABR, did not permit a direct measure of the tonotopic spread of excitation. Nevertheless, based on the marked difference in the slopes of eABR amplitude growth, we infer that IN stimulation gave a more restricted tonotopic spread. That inference comes from studies in cats [29] and humans [30] showing that CI stimulus configurations that elicited narrower spreads of excitation gave shallower growth of eABR slopes. The IN electrode that was used in the present study was larger in surface area than the silicon-substrate devices used in the 2007 study [8] and might be thought to be less selective. Performance was at least qualitatively equivalent between the two types of IN electrodes, however, in measures of threshold, dynamic range, and (inferred) spread of excitation.

In our previous study of transmission of TFS, we stimulated through IN or CI electrodes with pulse trains of varying rates and recorded synchronized spike activity in the ICC [10]. At pulse rates ~ 300 pps and higher, more than twice the percentage of ICC neurons synchronized to IN compared to CI stimulation. In that study, group delays of ICC neurons were ≥ 4 ms. In the present study, we interpreted eFFR group delays ≥ 4 ms as being dominated by a midbrain generator, probably the ICC. Midbrain sync cutoffs, then, were defined as the highest stimulus pulse rates at which the eFFR group delay was ≥ 4 ms. The present results confirmed the anticipated higher-rate temporal transmission available from IN stimulation. That is, the stimulation rate of 300 pps largely segregated midbrain sync cutoffs to > 300 pps for IN stimulation (mean across all days: 367 pps) versus < 300 pps for CI stimulation (mean: 241 pps). Subsequent terminal acute measures in a subset of cats replicated the higher midbrain sync cutoffs for IN compared to CI stimulation.

### The IN Electrode

A major challenge for long-term IN implantation and stimulation is to design a microelectrode that can deliver the needed electrical charge without causing electrochemical toxicity of the neural tissue or degradation of the electrode surface (reviewed in [31]). Charge density for IN microelectrodes is necessarily higher than for the macroelectrodes in a CI electrode array because of the difference in surface area: ~ 5000 µm^2^ for the IN electrodes versus 110,000 µm^2^ for the CI electrodes in the present study. Iridium oxide electrodes similar in design to our IN electrodes have a reported safe charge-injection capacity of ≥ 1 mC/cm^2^ [32]. The maximum safe charge-injection capacity of iridium oxide contrasts with that of platinum, only 0.1–0.2 mC/cm^2^, which is the material used for CI electrodes in clinical devices and in our animal CI arrays. That the in situ impedances of the IN and CI electrodes in the present study did not differ significantly (see the “Methods” section) indicates that the high charge-injection capacity of iridium oxide compensated adequately for the reduced surface area of the IN electrodes.

One example of the safety of iridium-oxide shanks for clinical application comes from tests of the penetrating auditory brainstem implant (PABI), which consists of an array of 8 to 10 shanks implanted in the human cochlear nucleus, each shank very similar to the IN electrodes in the present study [33]. Despite disappointing audiological results, the PABI patients showed no indication of damage to the nearby tissue or the electrodes over ~ 4 years of testing.

One goal of the present study was to test the safety and functional stability of IN stimulation for up to 6 months after implantation. We anticipated that degradation of auditory nerve function, if present, would manifest as gradual elevation of thresholds and/or lowering of midbrain sync cutoffs. We had seven cases of successful implantation of IN electrodes, as defined by measures in test sessions out to ~ 70 days after implantation. Four of the 5 IN-only cases made it to the target longevity of 6 months. The fifth IN-only case (Cat SC) failed ~ 90 days post-op due to fouling of the skull-mounted connector. There were 4 CI + 1 devices in which the IN components worked initially. Two of those devices worked properly through day 155 (FO) or 177 (BR) and then suffered a connector failure. The IN electrodes in the other 2 CI + 1 devices failed either due to movement while securing the device after implantation (Cat ED) or, apparently, due to migration of the IN electrodes to a non-favorable location in the nerve (Cat AB, day 73). Notably, neither of those failed CI + 1 cases showed changes in CI performance during the period of failing IN performance, indicating that there was no wholesale loss of nerve function. From that perspective, none of the seven successful IN implantations (five IN-only and two in CI + 1) showed evidence of degraded nerve function. Our working hypotheses for the failed IN implantations in CI + 1 Cats AB and ED are that the CI blocked the flow of cement to the IN electrode or that the larger bulla opening needed for the dual implantation permitted movement of the hardened block of cement. These are species-specific technical difficulties that will need to be overcome in our cat model, but they probably are not strongly relevant to translation of the CI + 1 to use in humans, which will no doubt present its own technical challenges.

### Maintenance of Temporal Acuity by Electrical Stimulation

We have shown previously that adult cats that were deafened and left unstimulated for ~ 6 months showed a profound loss of temporal acuity [13]. In that study, after 6 months of deafness, cats were anesthetized and implanted acutely with IN or CI electrodes. Thresholds and dynamic ranges for spike activity in the ICC showed little effect of 6 months of deafness despite a ~ 30% loss of auditory-nerve fibers seen in histology. Nevertheless, both IN and CI stimulation showed marked decreases in synchronized spike activity in the ICC. For instance, ~ 37% of ICC units fired synchronously to IN pulse trains at 300 pps in acutely deafened animals, whereas only ~ 2.4% synchronized to 300-pps IN pulse trains in 6-month deafened animals.

In that report [13], we marveled that human CI users can be as successful as they are in light of the profound loss of temporal acuity that we observed in deafened cats. We speculated that loss of temporal acuity in deaf humans is mitigated by auditory-pathway activity that is restored by users’ everyday use of their cochlear implants. In the present study, each animal was deafened as an adult and then received only about 1–2 h of electrical cochlear stimulation every 2–3 weeks, out to 6 months of implantation. In those conditions, there was no indication of loss of temporal acuity for either IN or CI stimulation, as shown by the absence of significant changes in midbrain sync cutoffs across as much as 6 months of testing. For that reason, we suggest that intermittent electrical stimulation was sufficient to ward off the sort of degradation of the auditory pathway that we observed in the previous study of total auditory deprivation.

Shepherd and colleagues [34] studied neonatal deafening in cats followed by acute cochlear implantation and unit recording from the ICC. In that study, bilateral deafening produced a marked loss of temporal acuity, whereas temporal acuity largely survived unilateral deafening even when tested with electrical stimulation of the deaf ear contralateral to the ICC recording site. That result suggests that unilateral acoustic stimulation is sufficient to maintain temporal acuity bilaterally. Here, we deafened adult cats bilaterally, and temporal acuity of the eFFR was maintained by intermittent unilateral electrical stimulation. That acuity was degraded, however, > 80 days after cessation of the intermittent stimulation in tests of acute stimulation of the non-stimulated ear (Fig. 11), although that loss of acuity was not as profound as that observed in ICC spike activity after ~ 6 months of bilateral deafness with no electric stimulation [13].

There is a body of literature that has examined effects of chronic electrical stimulation on temporal acuity in neonatally deafened cats. Early deafness resulted in loss of phase-locked spike activity in the ICC; e.g., [21, 34–36]. Remarkably, restoration of auditory nerve activity by electrical stimulation could promote fairly robust temporal acuity. In one study [36], cats that were deafened as neonates and tested as adults after receiving no chronic stimulation showed significantly degraded temporal acuity. In contrast, deafened cats that received some weeks of chronic stimulation beginning > 2.5 years after deafening showed temporal acuity that was, remarkably, even higher than that in normal-hearing controls. This and other studies with neonatal deafening show that chronic stimulation can enhance temporal acuity in animals that were deprived of patterned auditory activity during development. That is arguably a greater challenge than in the present study in which intermittent stimulation appears to have maintained temporal acuity in auditory pathways that had undergone normal development and then were deafened and began receiving intermittent stimulation after only 2–3 weeks of deafening.

### Limitations of Intrascalar Cochlear Implants for Apical Stimulation

The IN electrode in the present study targeted the central axons of apical spiral-ganglion cells. Rather than a penetrating electrode, one might consider stimulation of apical fibers using a deeply inserted intrascalar array. Indeed, deep-insertion arrays are produced commercially by Med-El [37] and other deep-insertion arrays have been tested by Cochlear Corporation [38]; there also is preliminary evidence that more-apical stimulation can be achieved by implanting a return electrode in the helicotrema [39]. Nevertheless, selective stimulation of apical fibers with an intrascalar device raises challenges beyond the mechanics of the deep insertion: in particular, non-specific tonotopic spread of excitation from an apical intrascalar electrode could disrupt transmission of TFS. The targets of intrascalar CI stimulation are the peripheral processes, cell bodies, and, to a lesser degree, central axons of the spiral ganglion. The spiral ganglion, however, extends only to the basal ~ 2 turns of the human cochlea, whereas the organ of Corti extends ~ 2–3/4 turns [40, 41]. Consequently, in humans, the apical 40% of the organ of Corti maps onto only the apical 20% of the spiral ganglion [40]. In the cochlear apex, both the spiral ganglion cells and their central axons are bunched together near the central axis of the cochlear spiral. In normal hearing, the tonotopic projection of the organ of Corti onto spiral ganglion neurons is accomplished by the trajectories of the ganglion-neurons’ peripheral processes. In electric hearing, those trajectories in the apex can distort the relationship between stimulated electrode and neuronal CF [42]. Moreover, gradual loss of peripheral processes is an expected sequela of loss of hair cells.

The challenges of achieving precise tonotopic stimulation of the apex are evident in place-pitch judgements by users of deep-insertion arrays. Those users often exhibit confusions of place-pitch sensations such that adjacent electrodes cannot be discriminated on the basis of pitch or that pitch reports are reversed from the expected high-to-low ranking corresponding to basal-to-apical electrode locations [38, 43–48]. A recent study of the mechanics of the apical cochlear in guinea pig has called into question the classical view of tonotopy at the apex [49], which could account to some degree for the difficulties in place pitch judgements with deep-insertion implants. Nevertheless, the reported confusions of place pitch sensation suggest limitations in the ability of apical intrascalar electrodes to stimulate populations of apical fibers selectively.

Studies of temporal acuity by apical stimulation with intrascalar CIs have produced mixed results. For example, low-rate stimulation of the most apical electrode of the deep-insertion MedEl device can produce better rate discrimination and “cleaner” sound sensations compared to stimulation of a mid-array electrodes [46, 50], but the upper limit of temporal pitch perception does not differ between the apical and mid-array sites [48].

Recent animal studies have explored optogenetic stimulation of spiral ganglion cells as an alternative to electrical stimulation [51]. In those studies, light-sensitive proteins are expressed in spiral ganglion cells and optical stimulation is delivered to desired cochlear loci. In principle, optical spread of cochlear stimulation can be more tonotopically restricted than electrical stimulation. Indeed, a study in gerbils showed cochlear spread of activation that was similar between optogenetic and pure-tone acoustical stimulation [52]. Optogenetic stimulation might someday offer more restricted stimulation of basal and middle turns in humans. Nevertheless, it is difficult to imagine how such stimulation could overcome the problem that the cell bodies of apical fibers are closely aggregated at the apex of the spiral ganglion lying ~ 3/4 of a turn basal to the apex of the organ of Corti.

### A Translational CI + 1 Device

We are working toward translation of the CI + 1 device to clinical trials. A CI + 1 for clinical use could consist of a conventional multi-electrode array inserted in the basal and middle turns of the scala tympani and a single iridium oxide IN electrode inserted in the trunk of the auditory nerve, targeting fibers from the cochlear apex. The IN electrode would offer two enhancements to the conventional cochlear prosthesis. First, stimulation of apical fibers with an IN electrode would extend dramatically the low-frequency end of the tonotopic range that could be stimulated selectively. The more-important enhancement, we think, would be enhanced transmission of TFS. That would be accomplished by stimulation of low-frequency brainstem pathways that are specialized for high temporal acuity, as demonstrated here and in our previous report [10]. The stimulation strategy for the CI + 1 would be a hybrid. The CI presumably would receive the conventional speech processing strategy consisting of constant high-rate pulse trains interleaved among electrodes and modulated by the envelopes of multiple bandpass filters. The IN electrode would receive trains of single pulses or of periodic brief bursts of pulses that would be synchronized to the TFS of low-frequency sounds or to an extracted fundamental frequency.

There are two cochlear prosthetic approaches in present clinical application that employ conventional stimulation with an intrascalar array in conjunction with preservation of TFS information at more apical cochlear sites. One very-successful approach is Electric Acoustic Stimulation (EAS), which can be used with patients who have intact low-frequency hearing (reviewed by [53]). Those patients receive a multi-electrode CI that transmits information from middle and high frequencies using conventional amplitude-modulated constant-rate pulse trains. Low frequencies are presented to the implanted ear as acoustic signals to the intact low-frequency cochlea. The benefits of the low-frequency component of EAS are most evident as improvements in speech reception in the presence of competing talkers (e.g., [54, 55]). Also, EAS users show improvements in pitch ranking and melody recognition compared to CI-only users [56, 57]. The principal limitation of EAS is, of course, that it requires that patients have residual low-frequency hearing, and for this to be preserved following implantation. In patients that lack sufficient acoustic hearing for successful EAS, IN stimulation with a CI + 1 might provide an alternative method for apical stimulation.

The second approach that combines a conventional CI with a strategy for temporal stimulation of the apical cochlea is the Fine Structure Processing (FSP) introduced by the Med-El company (Innsbruck, Austria) [58]. That strategy sends electrical TFS-locked pulse sequences to the most apical 1–4 electrodes of a deeply inserted CI. Performance varies rather widely among users and studies, with a recent review concluding that there was no convincing evidence for an improvement in speech perception [59]. Moreover, pitch percepts can depend markedly on the relative delays among the apical electrodes used for the FSP strategy [60]. We would argue that performance of FSP with today’s intrascalar arrays is hampered by the failure to achieve stimulation of restricted apical pathways. Nevertheless, some form of explicit coding of temporal fine structure will be called for as a strategy for stimulation of the IN component of the CI + 1.

We are optimistic for the future of IN stimulation in general as a solution to the limited temporal acuity that is available with today’s cochlear implants. The CI + 1 design is particularly well suited for translation to early clinical trials for the reason that, if the + 1 IN stimulation turned out not to work as promised, the remaining CI would leave the patient with essentially all the benefits of today’s cochlear implant. Success of IN stimulation, however, would allow clinicians to explore, and patients to enjoy, the potentially substantial benefits of selective apical stimulation in real-world application.

## Data Availability

Data are available from the corresponding author upon reasonable request.
